# Evaluating Bone Health in Egyptian Children with Forearm Fractures: A Case Control Study

**DOI:** 10.1155/2016/7297092

**Published:** 2016-08-29

**Authors:** Abeer El-Sakka, Cristina Penon, Adham Hegazy, Salwa Elbatrawy, Amr Gobashy, Alvaro Moreira

**Affiliations:** ^1^Department of Pediatrics, Ain Shams University Medical School, Cairo, Egypt; ^2^Department of Pediatrics, University of Texas Health Science Center, San Antonio, TX, USA; ^3^Department of Biology, National Research Center, Cairo, Egypt

## Abstract

*Objective*. To determine the likelihood of vitamin D deficiency and low bone mineral density in Egyptian children with forearm fractures.* Methods*. A case control study of 46 children aged 3 to 10 years with or without forearm fractures. Validated questionnaires were used to assess calcium and vitamin D intake as well as sun exposure. Serum calcium, phosphorus, alkaline phosphatase, and 25-hydroxy-vitamin D were collected. Bone mineral density was evaluated using dual-energy X-ray absorptiometry.* Results*. Compared to the Control group, calcium and vitamin D intake was lower in the Cases group (*p* = 0.03). Cases had higher Body Mass Index than Controls, *p* = 0.01. Children in the Cases group had lower mean serum calcium values 8.3 ± 1.4 compared to 9.3 ± 1.1 in Controls (*p* = 0.01). Alkaline phosphatase was higher in Cases 265 ± 65.8 than Controls 226 ± 54.6 (*p* = 0.03). Vitamin D and bone mineral density scores were significantly lower in the Cases group (*p* < 0.05).* Conclusion*. Our data shows an increased rate of vitamin D deficiency and decreased bone mineral density in Egyptian children with forearm fractures.

## 1. Introduction

Forearm fractures are the most common childhood fracture that presents to the emergency room in the United States [1]. Despite improvements in many childhood conditions, the incidence of forearm fractures in pediatric patients has increased throughout the last few decades. More concerning, children with a history of a forearm fracture have a higher risk of entering adulthood with decreased peak bone mass [2]. Although most research efforts have focused on fractures in late adulthood, there is emerging evidence that fractures in childhood may be the initial insult that leads to lifelong bone fragility [3].

In neonates and children, vitamin D deficiency has been directly linked to suboptimal bone mineralization and adverse conditions such as rickets and short stature [4–6].

Specifically, vitamin D is an important regulator of calcium (Ca) and phosphorus (P) homeostasis, the minerals necessary for bone formation [7]. Through its interactions in the kidney and intestine, activation of vitamin D stimulates bone mineralization [8, 9]. Furthermore, by the time children enter adolescence 40% of their peak bone mass has been accrued [10].

Dual-energy X-ray absorptiometry (DXA) imaging is a widely available clinical tool used in adults to diagnose osteoporosis, predict fracture risk, and monitor response to therapy [11]. With increased concern for future bone health, investigations using DXA in the pediatric population are becoming an area of high research interest [12, 13]. Similar to adults, DXA is the preferred imaging modal to measure bone mineral density (BMD) and bone mineral content in children due to the precise results, minimally invasive approach, and little exposure to radiation [14].

Multiple studies have demonstrated an association between vitamin D deficiency and fracture risk in children [14–17]. However, few studies have correlated vitamin D deficiency with BMD, fracture risk, and laboratory markers of bone health [18]. To our knowledge, no study has evaluated vitamin D status, BMD, and serologic markers of bone health in children from a developing country presenting to the emergency room with a forearm fracture.

Therefore, the aim of this study is to determine the prevalence of vitamin D deficiency and its effects on BMD in Egyptian children presenting with forearm fractures. We hypothesize that Egyptian children with forearm fractures have lower serum 25-hydroxy-vitamin D levels and BMD* z*-scores compared to children without forearm fractures. We also examined the relationship between vitamin D and serologic markers of bone health.

## 2. Methods

### 2.1. Study Center and Subjects

This study was conducted at Ain Shams University Hospital (ASUH) in Cairo, Egypt.

The Ethical Committee of ASUH approved the study which was conducted between December 2012 and June 2013 which corresponds to winter and spring in Egypt. Children between the ages of 3 and 10 years who presented to the Emergency Room at ASUH and had a confirmed radiologic fracture to the radius, ulna, or both were included in the study as Cases. Controls were children who had forearm pain or trauma but did not have radiographic evidence of forearm fracture. They were matched for gender and age to Cases.

Children were excluded from the study if they had a previous history of fractures, prolonged immobilization, or chronic use of antiepileptic drugs or glucocorticoids. Children with chronic illnesses that may interfere with bone health such as kidney disease or intestinal malabsorption were also excluded.

### 2.2. Study Design

This was a prospective case control study in children with or without forearm fractures to determine their vitamin D status and its impact on BMD. Calcium and vitamin D intakes were assessed via a modified food questionnaire [19]. Intake data was categorized into adequate, suboptimal, and low intake using the US Department of Agriculture Nutrient Database for Standard Reference [20] (Table 1). Extent of sun exposure was also categorized into daily, weekly, or no sun exposure [21].

### 2.3. Dual Energy X-Ray Absorptiometry (DXA) Measurements and Anthropometrics

All total body BMD measurements were performed using a Hologic Discovery Ci/Wi bone densitometer (Hologic™, Bedford, Massachusetts, USA) by a certified radiology technologist. Measurement results were obtained using Hologic's internal pediatric data software which takes into account patient's height, weight, and gender. Reported values were given as standard deviation scores (*z*-scores). A* z*-score of zero was equivalent to the mean, while a* z*-score between −1 and +1.5 was equivalent to values of one standard deviation below and 1.5 standard deviations above the mean, respectively. Per the International Society for Clinical Densitometry, a* z*-score less than or equal to 2 is defined as “low bone mass or bone mineral density” [22]. All measurements were performed and analyzed by the same individual.

### 2.4. Serologic Markers of Bone Health

The following laboratory tests were obtained in all subjects:

1. *Serum Ca* was measured via colorimetric assay (O-cresolphthalein complexone method) using SP120 autochemistry analyzer (Spectrum Diagnostics™, Cairo, Egypt). Normative values were provided by the manufacturer and were between 9.2 and 11 mg/dL in children of 4–16 years and 7.2 and 11.2 mg/dL in children of 4 weeks–3 years.
2. *Serum P* was measured by one-step colorimetric endpoint method without deproteinization by Vitro Scient Company™, Cairo, Egypt. Expected values were 4.4–5.5 mg/dL in children of 2–16 years.
3. *Serum alkaline phosphatase* (ALP) was measured by one-step colorimetric endpoint (Teco Diagnostics™, Anaheim, CA, USA). Coefficient of variance for this test is 3.1% to 4.2%. Normal values stated by the manufacturer include 32–92 IU/L.
4. *Serum vitamin D* measurements were analyzed using 25-hydroxy-vitamin D (^125^I RIA kit by DiaSorin Company™, Still Water, MN, USA). This assay uses direct competitive chemiluminescence using coated magnetic microparticles. Coefficient of variance for intraruns using this test is between 4.8% and 7.7%. The laboratory reference range is 30–100 ng/mL.

### 2.5. Vitamin D Categorization

Categorization of 25-OH-vitamin D results followed the recommendations from the American Academy of Pediatrics [23]:

- Sufficiency >20–100 ng/mL.
- Insufficiency >15–20 ng/mL.
- Deficiency ≤15 ng/mL.
- Severe Deficiency ≤5 ng/mL.

### 2.6. Statistical Analysis

Continuous data was analyzed using Student's* t*-test, and categorical data was analyzed using chi-squared analysis, or Fisher's exact test where appropriate. A *p* value ≤ 0.05 was considered statistically significant. STATA v.13 (Microsoft Corporation™, College Station, Texas, USA) was used to analyze data.

## 3. Results

### 3.1. Subjects

A total of 46 children were enrolled during the study period. There were 23 patients with confirmed forearm fractures in the Emergency Room at ASUH. The mean age was 7.2 ± 2.0 years, and 61% of subjects were boys. The demographic data is summarized in Table 2.

### 3.2. Calcium and Vitamin D Intake and Sun Exposure

Overall, half of the participants in the study had suboptimal or low intake of vitamin D and Ca. However, 30% of the subjects in the Cases group reported low vitamin D and Ca intake compared to 4% in the Controls group (*p* ≤ 0.05). Ninety-one percent of subject had reported either daily or weekly sun exposure. Four children with no reported sun exposure had forearm fractures (Table 2).

### 3.3. Calcium, Phosphorus, Alkaline Phosphatase, and Vitamin D Levels

Values for all serum Ca labs drawn ranged from 5.5 to 11.3 mg/dL with a mean of 8.8 mg/dL. Serum ALP levels drawn ranged from 160 to 400 IU/L, with a mean of 245 IU/L. Children with fractures had statistically significant lower serum Ca values (*p* ≤ 0.05) and higher serum ALP levels when compared with the Controls group (*p* ≤ 0.05). Vitamin D measurements ranged from 1.3 to 100 ng/mL with a mean of 38 ng/mL. Our study had one patient that was vitamin D deficient and eight patients with severe vitamin D deficiency. The four subjects in the Cases group with no reported sun exposure all had severe vitamin D deficiency. Data is summarized in Table 3.

### 3.4. Bone Mineral Density

**Figure 1:**
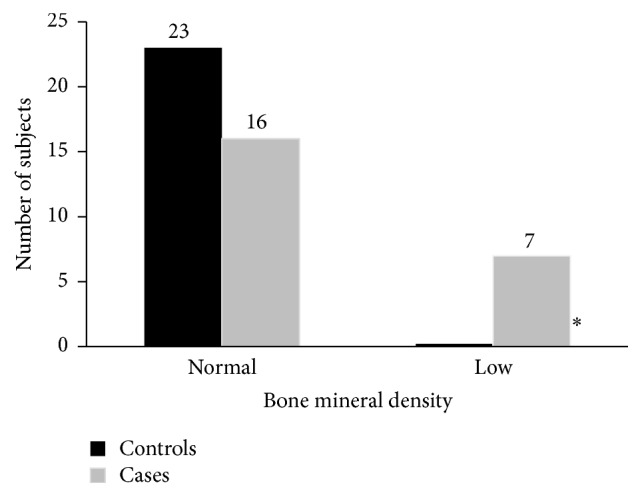
Bone mineral density in Cases and Controls. *∗* signifies* p* value ≤ 0.05.

The mean* z*-score in Cases was −0.19 ± 1.99 and 0.3 ± 1.04 in the Controls group (*p* = 0.3). The 7 Cases with severe vitamin D deficiency had a* z*-score ≤−2 (low bone mineral density). The single patient in the Controls group that had severe vitamin D deficiency had a* z*-score of −1.9. Graphic representation is seen in Figure 1.

### 3.5. Bone Serologic Markers in Relation to Vitamin D Level

**Figure 2:**
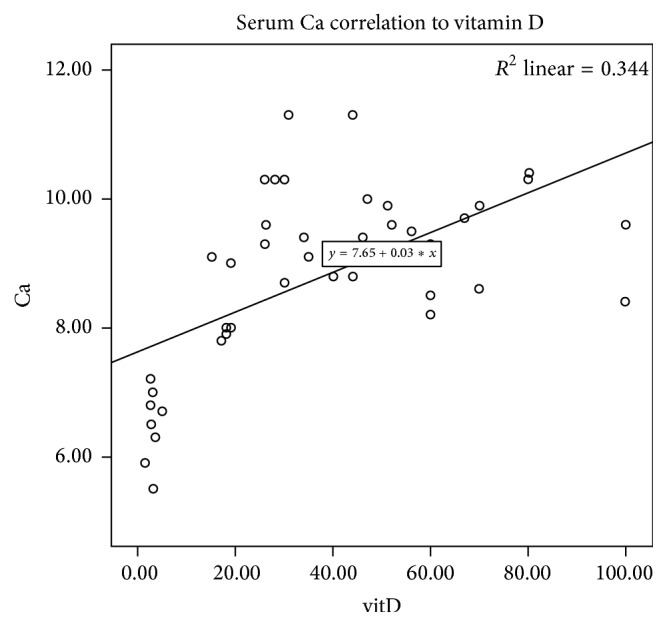
Serum calcium correlation to vitamin D. ^*∗*^ *R* ^2^ = 0.334, *p* < 0.01.

As shown in Figure 2, serum Ca had correlations with serum 25-OH-vitamin D levels (*p* ≤ 0.01). There were no significant correlations found between serum 25-OH-vitamin D and phosphorus or alkaline phosphatase. No association was found between Vitamin D and gender, age, or Body Mass Index (BMI).

## 4. Discussion

This study found a high percentage of severe vitamin D deficiency in Egyptian children with forearm fractures. Furthermore, this study demonstrates that 25-OH-vitamin D is an important marker of bone mineralization in children with fractures.

Our findings are in agreement with studies by Ryan et al. [17, 18] who found that a significant proportion of children with forearm fractures were vitamin D insufficient [21, 22]. In our study, 33% of children had vitamin D insufficiency. Similarly, their studies describe a direct association between 25-OH-vitamin D levels and BMD* z*-scores. The patients in our study had overall lower* z*-scores compared to the subjects in their study (.06* z*-score versus 0.8* z*-score). Collecting dietary calcium and vitamin D intake and sunlight exposure allows us to infer that the differences in* z*-scores may be secondary to the suboptimal/low nutritional status (50%) of the Egyptian children, as well as the lack of sun exposure [24, 25].

Vitamin D synthesis from the sun is considered an important source of vitamin D [26]. Our study included four individuals with no sun exposure who were found to have vitamin D deficiency as well as low BMD measurements. This highlights the importance of sun light exposure in bone health in children. This was recently emphasized by studies comparing indoor and outdoor training in athletes and found significant lower Vitamin D levels in those with indoor training [27]. Our study adds to the growing literature that supports the importance of outdoor sunlight exposure for children. Studies as early as the industrial revolution in Europe have demonstrated bone deformities in children living in the inner cities exposed to minimal sun exposure [28].

This study showed a significant correlation between serum calcium levels and vitamin D. Vitamin D plays an important role in calcium homeostasis through its absorption in the intestine and kidney. In cases of poor calcium intake or absorption, 1,25-hydroxyvitamin D mobilizes Ca from bone thereby inhibiting mineral deposition into the osteoid matrix [29, 30]. In postmenopausal women, low levels of serum calcium are frequently associated with increased risk for fractures [31]. Furthermore, poor calcium intake and distal forearm fractures during childhood are strong predictors of skeletal fragility in adulthood [32, 33].

In agreement with our results Michałus et al. found lower mean values of BMD* z*-scores with higher alkaline phosphatase levels in children who had multiple bone fractures [34]. In our study, although serum ALP levels were within normal reference values, they were significantly higher in those with fractures. The importance of serum calcium and alkaline phosphatase as markers for abnormal bone health and osteomalacia has been described in the study by Peach et al. [35]. In their study, they found elevated plasma alkaline phosphatase and hypocalcemia in 48% of patients diagnosed histologically as having osteomalacia. Moreover, alkaline phosphatase measurements have recently been recommended as a screening tool for osteomalacia [36]. Also, ALP has been described as a screening tool for preterm newborns at risk for metabolic bone disease, especially when accompanied with low serum phosphate [37].

We found that Cases have significant higher Body Mass Index (BMI) than Controls, *p* = 0.01. This supports other studies which found that high adiposity is associated with increased risk of forearm fractures in children [38].

This study is important because children who develop vitamin D deficiency can potentially have life-long complications. Considering that peak bone mass is reached by the end of the second decade of life, children with early fractures have a high probability of future fracture and more concerning osteoporosis later in life [39, 40]. Thus improvement in awareness of bone health in the pediatric population can directly impact on quality of life. Although pubertal status is important in evaluating BMD, our subjects were in the age between 3 and 10 years which is less than the optimal age of puberty in Egyptian children [41].

A major limitation of this study is the small number of subjects included in the study. Larger studies are necessary to corroborate our findings.

## 5. Conclusions

This study demonstrates that vitamin D insufficiency/deficiency is common among Middle Eastern children. It points out the importance of assessing vitamin D status in children with fractures.

## Figures and Tables

**Table 1:** Calcium and vitamin D intake questionnaire.

Intake question (weekly basis)	Approximate calcium content (mg)	Approximate vitamin D content (IU)
How often does your child drink milk?	1 cup whole milk: 246	Vitamin D fortified cup of milk: 115–124
How often does your child eat…		
Yogurt?	6 ounces nonfat yogurt: 258	Fortified with 20% for daily value (DV)
Cheese?	1 ounce cheese: 202	1 ounce cheese: 6
Fish?		Salmon, cooked, 3 ounces: 794 2 sardines, canned in oil, drained: 46
Eggs?		1 whole egg: 25
Liver?		Liver, beef, cooked, 3.5 ounces: 46
Does your child take Ca or vitamin D supplementation?	Varies	Varies

Questionnaire modified from Greer et al. [19]. Approximations obtained from US Department of Agriculture Nutrient Data Laboratory [20].

**Table 2:** Subject characteristics and calcium and vitamin D intake.

	Controls (*n* = 23)	Cases (*n* = 23)	*p* value
*Subject characteristics*			
Age, years	7.7 ± 1.7	6.7 ± 2.3	0.08
Weight, kg	25.6 ± 5.1	22.3 ± 6.0	0.06
Height, cm	123.7 ± 10.1	116.8 ± 13.3	0.06
Male gender	14 (60.8)	14 (60.8)	1
Body mass index (kg/m^2^)	16.69 ± 0.94	15.64 ± 1.84	0.01
Calcium and vitamin D intake			0.03^*∗*^
Adequate	11 (47.8)	11 (47.8)	
Suboptimal	11 (47.8)	5 (21.7)	
Low	1 (4.3)	7 (30.4)	
Sun exposure			0.09
Daily	15 (65.2)	10 (43.5)	
Weekly	8 (34.8)	9 (39.1)	
None	0	4 (17.4)	

Results are expressed as mean ± standard deviation or *n* (%).

*∗* signifies *p* value ≤ 0.05.

**Table 3:** Vitamin D status and serum calcium, phosphorus, and alkaline phosphatase levels.

	Controls (*n* = 23)	Cases (*n* = 23)	*p* value
Calcium, mg/dL	9.3 ± 1.1	8.3 ± 1.4	0.01^*∗*^
Phosphorus, mg/dL	4.0 ± 0.7	3.7 ± 0.8	0.21
ALP, IU/L	226 ± 54.6	265 ± 65.8	0.03^*∗*^
25-OH vitamin D categorized			0.047^*∗*^
Sufficient	18 (78.3)	13 (56.5)	
Insufficient	4 (17.4)	2 (8.7)	
Deficiency	0 (0)	1 (4.3)	
Severe	1 (4.3)	7 (30.4)	

Results are expressed as mean ± standard deviation or *n* (%).

*∗* signifies *p* value ≤ 0.05.
